# Plasma metabolomic profile associated with fatigue in cancer patients

**DOI:** 10.1002/cam4.3749

**Published:** 2021-02-03

**Authors:** Li Rebekah Feng, Jennifer J. Barb, Jeniece Regan, Leorey N. Saligan

**Affiliations:** ^1^ National Institute of Nursing Research National Institutes of Health Bethesda MD USA; ^2^ Clinical Center National Institutes of Health Bethesda MD USA; ^3^ The Pennsylvania State University College of Medicine Hershey PA USA

**Keywords:** cancer fatigue, cancer‐related fatigue, fatigue, metabolomics

## Abstract

**Background:**

Metabolomics is the newest ‐omics methodology and allows for a functional snapshot of the biochemical activity and cellular state. The goal of this study is to characterize metabolomic profiles associated with cancer‐related fatigue, a debilitating symptom commonly reported by oncology patients.

**Methods:**

Untargeted ultrahigh performance liquid chromatography/mass spectrometry metabolomics approach was used to identify metabolites in plasma samples collected from a total of 197 participants with or without cancer. Partial least squares‐discriminant analysis (PLS‐DA) was used to identify discriminant metabolite features, and diagnostic performance of selected classifiers was quantified using area under the receiver operating characteristics (AUROC) curve analysis. Pathway enrichment analysis was performed using Fisher's exact test and the Kyoto Encyclopedia of Genes and Genomes (KEGG) metabolic pathway database.

**Findings:**

The global metabolomics approach yielded a total of 1120 compounds of known identity. Significant metabolic pathways unique to fatigued cancer versus control groups included sphingolipid metabolism, histidine metabolism, and cysteine and methionine metabolism. Significant pathways unique to non‐fatigued cancer versus control groups included inositol phosphate metabolism, primary bile acid biosynthesis, ascorbate and aldarate metabolism, starch and sucrose metabolism, and pentose and glucuronate interconversions. Pathways shared between the two comparisons included caffeine metabolism, tyrosine metabolism, steroid hormone biosynthesis, sulfur metabolism, and phenylalanine metabolism.

**Conclusions:**

We found significant metabolomic profile differences associated with cancer‐related fatigue. By comparing metabolic signatures unique to fatigued cancer patients with metabolites associated with, but not unique to, fatigued cancer individuals (overlap pathways) and metabolites associated with cancer but not fatigue, we provided a broad view of the metabolic phenotype of cancer‐related fatigue.

## Graphical abstract

We found significant metabolomic profile differences associated with cancer‐related fatigue. Potentially impactful metabolic pathways associated specifically with fatigued cancer patients include changes in metabolites associated with sphingolipid metabolism, histidine metabolism, and cysteine and methionine metabolism.

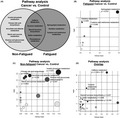

## 1 INTRODUCTION

Fatigue is a common symptom reported by most oncology patients and can be distinguished from tiredness experienced by healthy individuals as it is not relieved by sleep and is disproportional to exertion levels.^1^ Cancer‐related fatigue has a profoundly negative impact on the patient's quality of life and manifests both physically and cognitively.^2^, ^3^, ^4^, ^5^ Despite the burden cancer‐related fatigue places on patients, there is currently no well‐established biomarkers or FDA‐approved therapeutic interventions.^1^ As a result, cancer‐related fatigue has recently been identified by the National Cancer Institute as one of the top five high‐priority research areas.^6^ Fatigue phenotypes associated with different types of cancer may arise from various pathogenic processes.^1^ However, emerging evidence suggests that the common theme among these different etiologies may be related to intrinsic genetic vulnerability combined with inflammatory triggers related to cancer or cancer treatment.^2^, ^7^, ^8^, ^9^, ^10^

Metabolomics, or metabolome profiling, is the newest addition to the ‐omics methodology and refers to the global identification and quantification of metabolites.^11^ By definition, metabolites are small molecule metabolic products that result from chemical transformation during metabolism and, therefore, provide a real‐time functional snapshot of the biochemical activity and cellular state.^12^ Strategies for conducting metabolomics studies generally fall into two categories: “targeted” metabolomics refers to the methodology of measuring a limited number of chemically characterized and biochemically annotated metabolites; in contrast, the “untargeted”, or global, strategy detects as many distinct chromatographic features (e.g., mass‐to‐charge ratio) as possible and the identities of metabolites are subsequently determined using a reference spectral library.^13^ The development of new mass spectrometry techniques has allowed for instantaneous measurement of thousands of metabolites including amino acids, lipids, fatty acids, sugars, vitamins, as well as products of various metabolic processes.^14^ Given that information on tissue specificity and temporal dynamics can be difficult to derive from other types of ‐omics approach, it is not surprising that metabolomics has gained popularity in recent years as a powerful tool for biomarker discovery and understanding disease‐related metabolic pathways in complex diseases.^15^

Identification of metabolic signatures of cancer‐related fatigue is in the beginning stages and much remains to be explored to map out a complete metabolomic profile of this debilitating condition.^16^ With the advancement of new methodologies, it is now feasible to measure thousands of metabolites at the same time in an unbiased manner. The goal of this study is to perform a comprehensive metabolomic profile analysis using plasma samples from a total of 197 participants including those with confirmed cancer diagnosis and healthy controls. With the powerful and comprehensive untargeted LC‐MS approach, we identified novel metabolite markers and pathways specific to cancer‐related fatigue.

## 2 METHODS

### 2.1 Participants

Participants were enrolled from July 2009 to August 2019 at the National Institute of Health (NIH) Clinical Center, Bethesda, Maryland. The study was approved by the NIH Institutional Review Board (IRB). Signed, written informed consents were obtained prior to study participation. All study participants were male and ≥18 years of age. The exclusion criteria for healthy controls included severe psychiatric conditions, fatigue induced by clinically confirmed disease, or taking medication with a fatigue side effect. The cancer participants were from two clinical protocols: (1) the first protocol enrolled male participants who were scheduled to receive external radiation therapy (EBRT) at the NIH for localized prostate cancer; (2) the second protocol is a mixed cancer protocol including patients with confirmed cancer diagnosis (including non‐prostate cancer types) and a scheduled cancer treatment (including non‐EBRT). Exclusion criteria for both cancer protocols included a history of psychiatric disease in the last 5 years, a disease causing clinically significant fatigue, a history of tuberculosis, chronic inflammatory disease, uncorrected hypothyroidism, uncontrolled anemia, or a systemic infection. The difference between the two cancer protocols is the cancer type and scheduled cancer treatment. Samples from only the baseline (pre‐treatment) timepoint were included in the current study to avoid treatment‐related metabolic changes.

### 2.2 Instruments

Clinical data were obtained from chart review. Fatigue severity was measured using the 13‐item Functional Assessment of Chronic Illness Therapy – Fatigue (FACIT‐F), a validated, reliable, stand‐alone measure of cancer‐related fatigue (additional details: www.facit.org).^17^ FACIT‐F measures demonstrated good internal consistency reliability (Cronbach's *α* = 0.81) in our study cohort. Total FACIT‐F scores typically range from 16 to 53; lower scores indicate higher fatigue intensity. FACIT‐F required subjects to recall their fatigue experience in the past 7 days. A FACIT‐F score of 43 best differentiates fatigue scores of cancer patients from the general population.^18^ This method of fatigue classification employs cross‐sectional comparisons of the participant's FACIT‐F score with the mean score of the general population in the US.^18^ Participants were considered “fatigued” at a FACIT‐F < 43, and “non‐fatigued” at a FACIT‐F ≥ 43.

### 2.3 Metabolomics

Baseline (prior to cancer treatment) timepoint plasma samples were used in metabolomics analyses. Briefly, blood from each participant was drawn into an ethylenediaminetetraacetic acid (EDTA) tube (BD Biosciences), processed according to manufacturer's instructions, and frozen at −80°C until further analysis. Untargeted metabolomics analysis was performed at Metabolon, Inc., as described previously.^19^ Briefly, individual samples were subjected to methanol extraction and divided into aliquots for analysis using ultrahigh performance liquid chromatography/mass spectrometry (UHPLC/MS). Instrument variability was determined by calculating the median relative standard deviation (RSD) for the standards that were added to each sample prior to injection into the mass spectrometers. Overall process variability was determined by calculating the median RSD for all endogenous metabolites present in 100% of the pooled matrix samples. All methods utilized a Waters ACQUITY ultra‐performance liquid chromatography (UPLC) and a Thermo Scientific Q‐Exactive high‐resolution/accurate mass spectrometer interfaced with a heated electrospray ionization (HESI‐II) source and Orbitrap mass analyzer operated at 35,000 mass resolution. The global biochemical profiling analysis comprised of four unique arms consisting of reverse phase chromatography positive ionization methods optimized for hydrophilic compounds (LC/MS Pos Polar) and hydrophobic compounds (LC/MS Pos Lipid), reverse phase chromatography with negative ionization conditions (LC/MS Neg), as well as a hydrophilic interaction liquid chromatography (HILIC) method coupled to negative (LC/MS Polar). All of the methods alternated between full‐scan MS and data‐dependent multi‐stage mass spectrometry (MSn) scans. The scan range varied slightly between methods but generally covered 70–1000 m/z.

Peaks were quantified using area under the curve. Data normalization was performed to correct variation resulting from instrument inter‐day tuning differences. Metabolites were identified by automated comparison of the ion features in the experimental samples to a reference library of chemical standard entries that included retention time/index (RI), mass to charge ratio/molecular weight (*m*/*z*), preferred adducts, and in‐source fragments as well as associated MS spectra and curated by visual inspection for quality control. Identification of known chemical entities was based on comparison to metabolomic library entries of purified standards.^20^

### 2.4 Statistical analysis

Metabolite concentrations were normalized to sample volume utilized for extraction. Each metabolite was rescaled to set the median equal to 1. Normalized metabolite concentrations were logarithm base 10 transformed. Log‐transformed metabolomic data were analyzed using univariate analysis of variance (ANOVA) and *t*‐tests (unpaired, unequal variance assumed) to generate the volcano plots, and partial least squares‐discriminant analysis (PLS‐DA) was used to determine the variance importance in projection (VIP). Multiple comparisons were adjusted with the Benjamini‐Hochberg False Discovery Rate (FDR) method. Metabolites with VIP scores determined by PLS‐DA that were greater than 1.5 were considered significant.^21^ Leave‐One‐Out Cross‐Validation (LOOCV) and permutation tests were performed to test the model with *Q* ^2^ and *R* ^2^ being used to assess the robustness of the model and the amount of variation represented by the principal components and the permutation significance threshold set at *p* < 0.05. Metabolites were considered significant features for further analysis at VIP >1.5, |log2 fold change| >1.5, and false discovery rate ≤10%.^22^ Diagnostic performance of selected classifiers was quantified using the area under the receiver operating characteristics (AUROC) curve analysis. Metabolite pathway analysis using Fisher's exact test and the Kyoto Encyclopedia of Genes and Genomes (KEGG) metabolomics reference library was performed in MetaboAnalyst 4.0 as previously described.^23^ Statistical significance is defined as *p* < 0.05. Data analysis was performed using a combination of JMP™ Statistical Discovery Software 15 15.0.0 (SAS Institute) and MetaboAnalyst 4.0.^23^

## 3 RESULTS

### 3.1 Demographics

**FIGURE 1:**
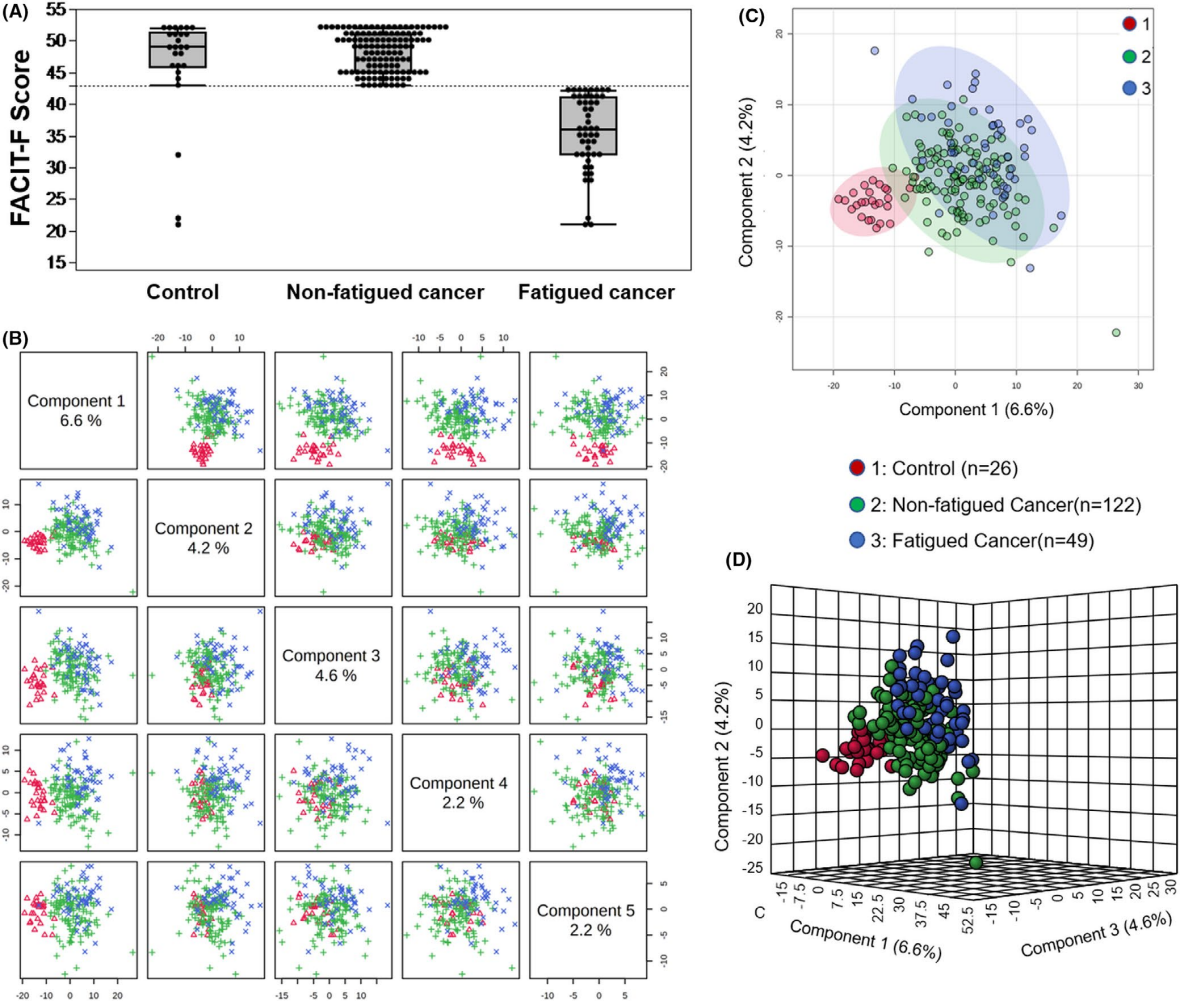
Metabolic profiles in fatigued and non‐fatigued cancer and healthy controls. (A) Box plot showing FACIT‐F scores of healthy controls (46.27 ± 8.45), non‐fatigued cancer (48.24 ± 3.02), and fatigued‐cancer groups (35.57 ± 5.76). Differences in FACIT‐F scores were significant between fatigued cancer versus controls (*p* = 1.168 × 10^−6^) and fatigued cancer versus non‐fatigued cancer (*p* = 2.929 × 10^−21^), but not between non‐fatigued cancer versus controls (*p* = 0.252). (B) Pairwise partial least squares‐discriminant analysis (PLS‐DA) score plot of the top five components demonstrating the separation of the groups. (C) PLS‐DA two‐dimensional plot ellipses representing 95% confidence intervals. (D) Three‐dimensional PLS‐DA plot showing model discrimination of fatigued cancer, non‐fatigued cancer, and healthy control groups

Participants were a total of 197 men with and without cancer (Table S1). Participants in the fatigued cancer (*n* = 49) and non‐fatigued cancer groups (*n* = 122) were older than healthy controls (*n* = 26) (65.35 ± 8.58 and 65.25 ± 8.55 vs. 35.92 ± 15.03 years) (Table S1). Since age did not correlate with FACIT‐F scores (*r* = −0.058, *p* = 0.421), the study did not exclude healthy controls who were significantly younger than the cancer group. Fatigued cancer patients had the highest body mass index (BMI) compared to non‐fatigued cancer patients and healthy controls (Table S1; fatigued cancer: 30.37 ± 5.34, non‐fatigued cancer: 27.85 ± 3.93, control: 26.81 ± 3.65). Cancer participants reported fatigue, defined as FACIT‐F score <43, at a greater percentage (28.65%) compared to healthy controls (11.11%). FACIT‐F scores of the fatigued cancer group were significantly lower than the healthy control group (fatigued cancer: 35.57 ± 5.76, control: 46.27 ± 8.45, *p* = 1.168 × 10^−6^) indicating higher fatigue severity; no difference was observed between non‐fatigued cancer and healthy control groups (non‐fatigued cancer: 48.24 ± 3.02, control: 46.27 ± 8.45, *p* = 0.252) (Figure 1A).

### 3.2 Metabolic signatures associated with cancer‐related fatigue

**FIGURE 2:**
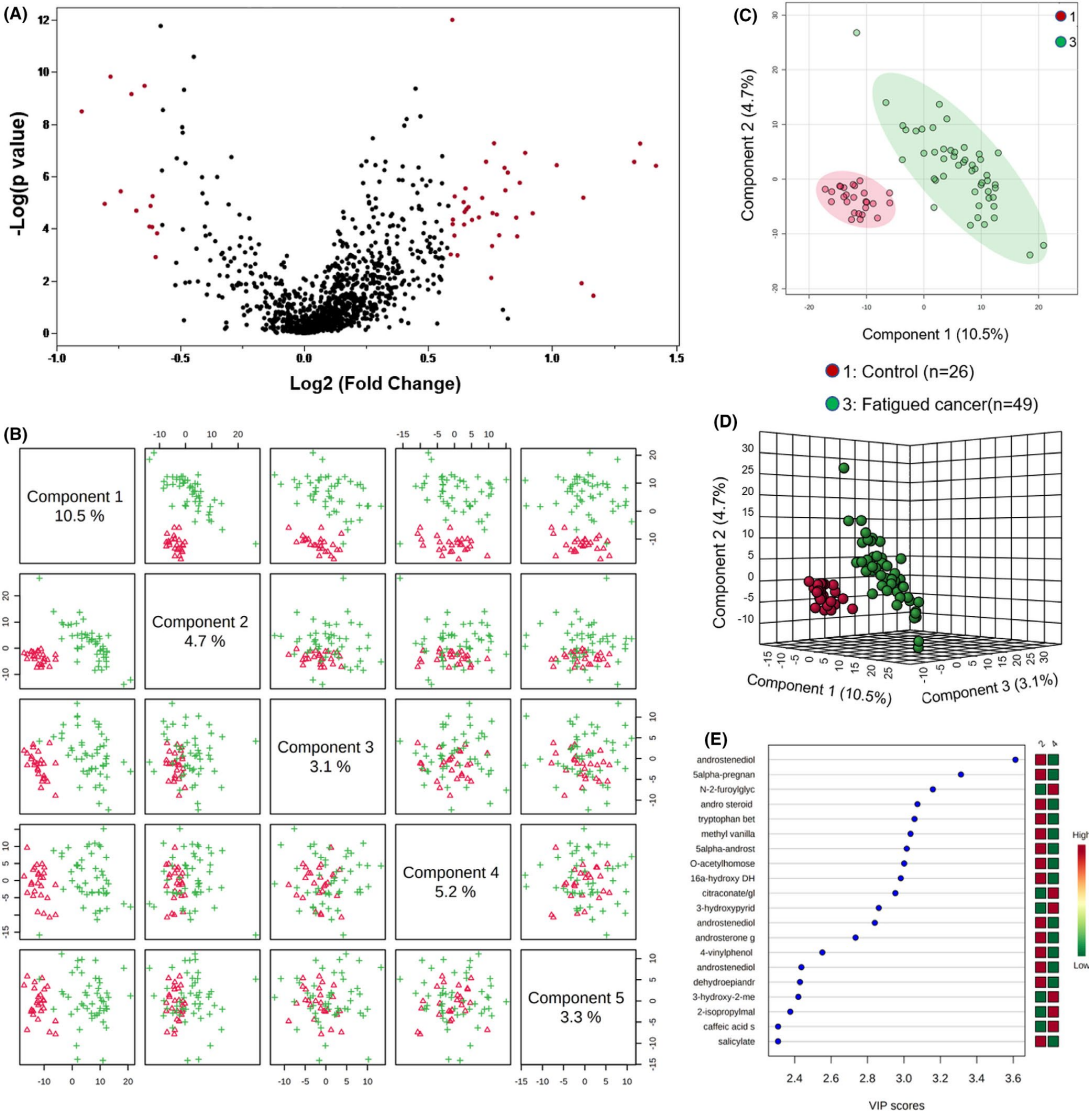
Metabolite profile of fatigued cancer patients compared to healthy controls. (A) Volcano plot of metabolites of fatigued cancer compared to fatigued healthy controls. The y‐axis represents p‐value converted to negative log 10 scale and the x‐axis represents log2 fold change. Significant metabolites (fold change >1.5, FDR ≤0.1) were highlighted in red. (B) Pairwise PLS‐DA score plot of the top five components demonstrating good separation between fatigued cancer patients and healthy controls. (C) PLS‐DA two‐dimensional plot ellipses representing 95% confidence intervals. (D) Three‐dimensional PLS‐DA plot showing good model discrimination between fatigued cancer patients compared to healthy controls. (E) Variable Importance in Projection (VIP) plot generated from the PLS‐DA analysis showing the most discriminative metabolites in descending order of importance

**FIGURE 3:**
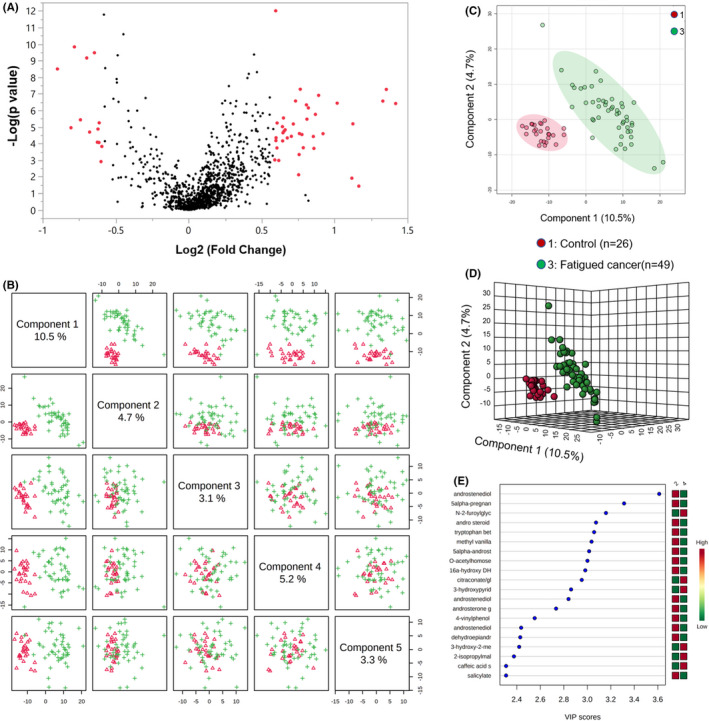
Metabolite profile of non‐fatigued cancer patients compared to healthy controls. (A) Volcano plot of metabolites of non‐fatigued cancer compared to non‐fatigued healthy controls. The y‐axis represents *p*‐value converted to negative log 10 scale and the *x*‐axis represents log2 fold change. Significant metabolites (fold change >1.5, FDR ≤0.1) were highlighted in red. (B) Pairwise PLS‐DA score plot of the top five components demonstrating good separation between non‐fatigued cancer patients and healthy controls. (C) PLS‐DA two‐dimensional plot ellipses representing 95% confidence intervals. (D) Three‐dimensional PLS‐DA plot showing model discrimination between groups. (E) VIP plot generated from the PLS‐DA analysis showing the most discriminative metabolites in descending order of importance

Untargeted LC/MS yielded a total of 1120 compounds of known identity including 459 lipids, 267 xenobiotics, 221 amino acids, 39 cofactors and vitamins, 39 peptides, 38 nucleotides, 28 carbohydrates, and 11 energy metabolism‐related metabolites. A supervised method of PLS‐DA distinguished fatigued cancer and non‐fatigued cancer groups from the controls (Figure 1B–D). Significant metabolite features that distinguished participants with cancer‐related fatigue from controls (metabolite list of fatigue cancer vs. control see Table S2) were selected based on a log2 fold change cutoff at 1.5 and 10% false discovery rate (Figure 2A), and a variable importance in projection (VIP) score of 1.5 as determined by PLS‐DA (Figure 2B–E). Despite the lack of difference in FACIT‐F scores, non‐fatigued cancer participants can be metabolically distinguished from healthy controls (Figure 3). Features of non‐fatigued cancer patients compared to healthy controls (metabolite list of non‐fatigue cancer vs. control see Table S3) were considered significant based on a log2 fold change cutoff of 1.5 at 10% false discovery rate (Figure 3A), and a PLS‐DA VIP score of ≥1.5 (Figure 3B–E).

**FIGURE 4:**
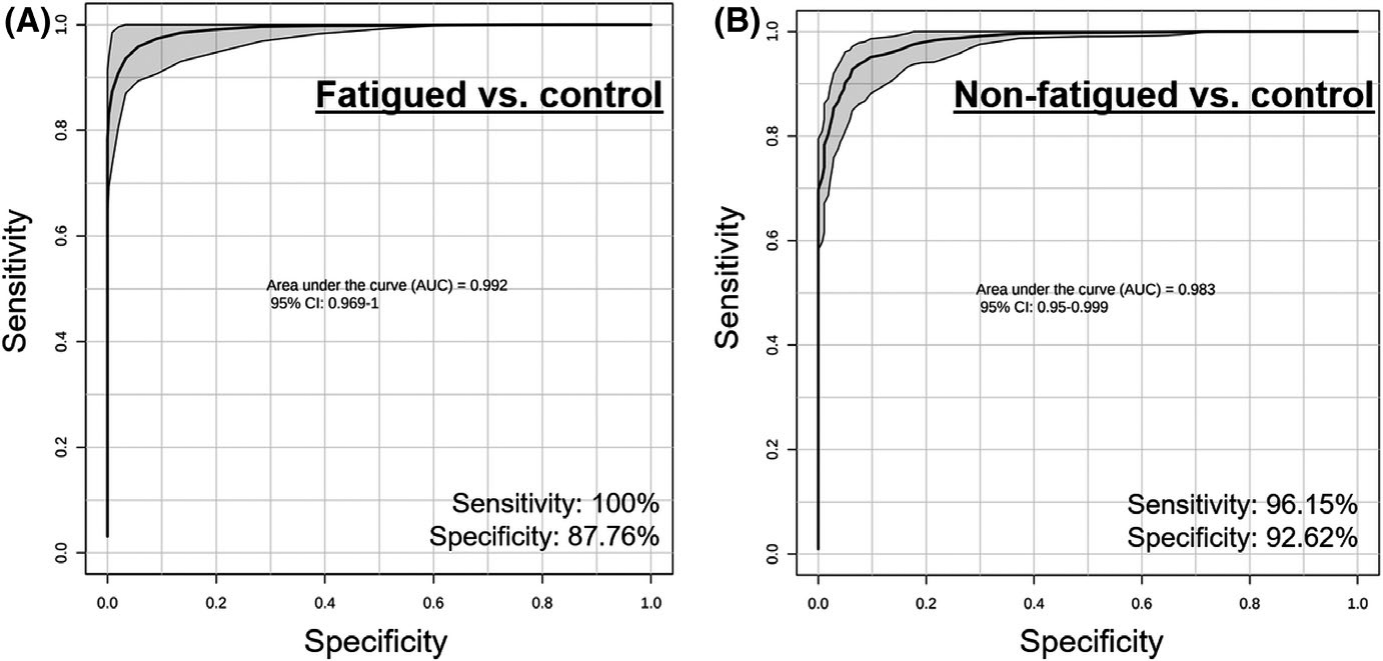
AUROC curve analysis of PLS‐DA model fit. (A) ROC curve showing the specificity and sensitivity of the PLS‐DA model (AUC = 0.992, 95% CI [0.969, 1]) demonstrating excellent classification capability of the model to distinguish fatigued cancer from controls. (B) ROC curve showing the specificity and sensitivity of the PLS‐DA model (AUC = 0.983, 95% CI [0.95, 0.999]) demonstrating excellent classification capability of the model to distinguish non‐fatigued cancer from controls. Both PLS‐DA models achieved excellent classification as indicated by an AUC > 0.90

Receiver operator characteristic (AUROC) curve analysis was performed to test the specificity and sensitivity of the models (Figure 4). Metabolites that distinguished fatigued cancer patients and controls (Figure 2; Table S2) demonstrated an AUC of 0.992 [95% confidence interval (CI) = 0.969, 1] with 87.76% specificity and 100% sensitivity (Figure 4A). ROC curve also showed good separation of non‐fatigued cancer patients and controls (Figure 3; Table S3) at AUC 0.983 (95% CI = 0.95, 0.999) with 92.62% specificity and 96.15% sensitivity (Figure 4B).

### 3.3 Metabolic pathways related to cancer‐related fatigue

**FIGURE 5:**
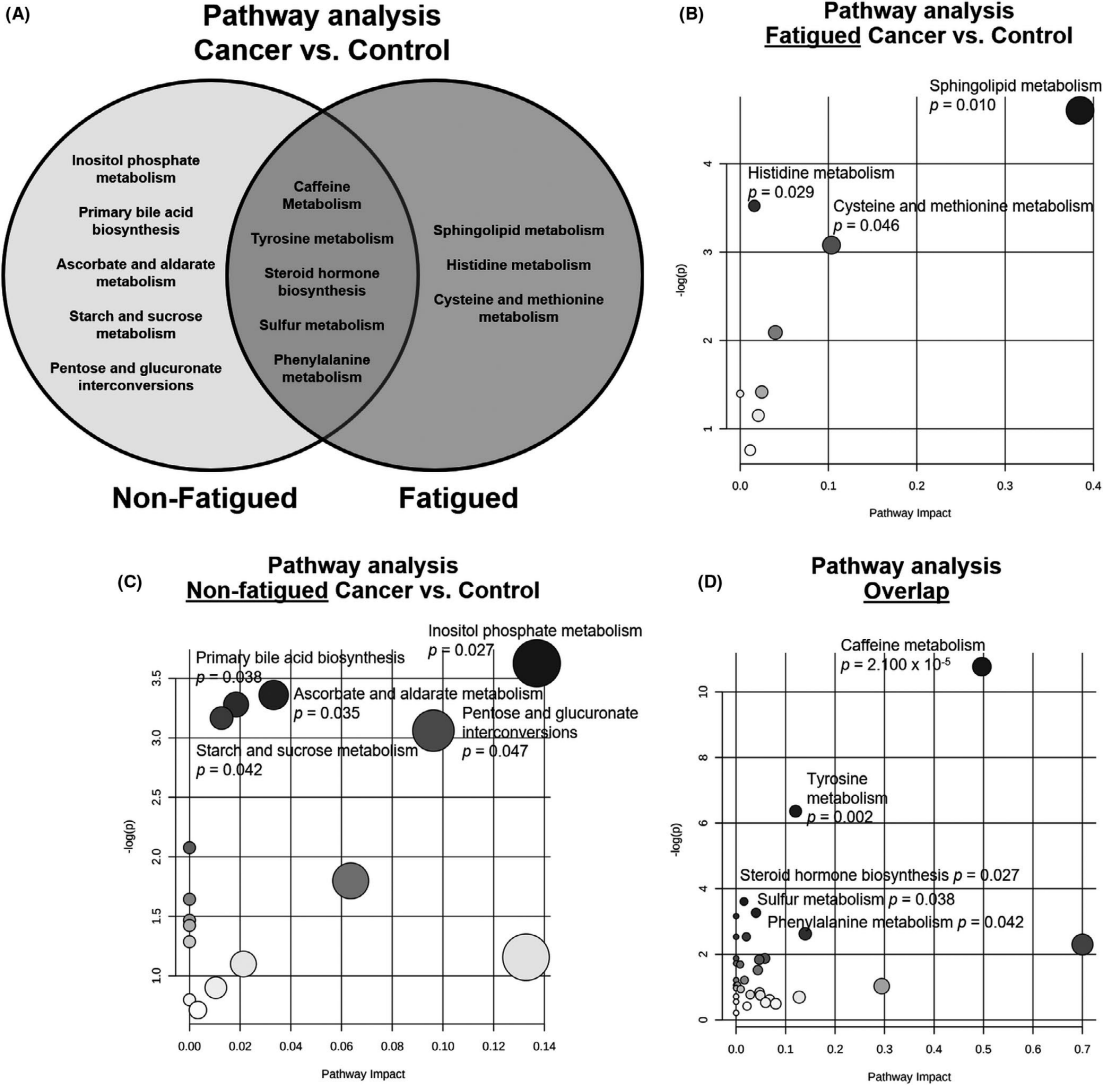
Metabolite pathway analysis using Fisher's exact test and the Kyoto Encyclopedia of Genes and Genomes (KEGG) metabolic pathway database. (A) Venn diagram of significant pathways unique to non‐fatigued (left), fatigued (right), and overlap between non‐fatigued and fatigued participants with cancer compared to controls. (B) Overrepresented pathways of metabolites that were only significantly different between fatigued cancer versus healthy controls. The x‐axis represents pathway impact, and the y‐axis represents ‐log(p). (C) Overrepresented pathways of metabolites that were only significantly different between non‐fatigued cancer versus healthy controls. The x‐axis represents pathway impact, and the y‐axis represents ‐log(p). (D) Overlap of overrepresented pathways between the two comparisons. The x‐axis represents pathway impact, and the y‐axis represents ‐log(p). Gray scale gradient and circle size indicate the significance of the pathway ranked by *p*‐value and pathway impact score. Statistically significant pathways are annotated (*p* < 0.05)

Discriminant metabolites unique to each comparison (fatigued cancer vs. control, non‐fatigued cancer vs. control), as well as those shared between the two comparisons, were used to identify overrepresented metabolic pathways (Figure 5A). Pathway enrichment analysis was performed using Fisher's exact test and the KEGG metabolic pathway database. Statistically significant pathways overrepresented by significant metabolites unique to fatigued cancer versus control groups included sphingolipid metabolism (*p* = 0.010), histidine metabolism (*p* = 0.029), and cysteine and methionine metabolism (*p* = 0.046) with sphingolipid metabolism at the highest pathway impact level and statistical significance (Figure 5B). Significant pathways overrepresented by metabolites unique to non‐fatigued cancer versus control groups included inositol phosphate metabolism (*p* = 0.027), primary bile acid biosynthesis (*p* = 0.038), ascorbate and aldarate metabolism (*p* = 0.035), starch and sucrose metabolism (*p* = 0.042), and pentose and glucuronate interconversions (*p* = 0.047) with inositol phosphate metabolism at the highest pathway impact level and statistical significance (Figure 5C). Metabolites that the two aforementioned comparisons shared in common were enriched in pathways including caffeine metabolism (*p* = 2.100 × 10^−5^), tyrosine metabolism (*p* = 0.002), steroid hormone biosynthesis (*p* = 0.027), sulfur metabolism (*p* = 0.038), and phenylalanine metabolism (*p* = 0.042) with caffeine metabolism at the highest pathway impact level and statistical significance (Figure 5D).

## 4 DISCUSSION

In this study, we utilized the untargeted LC/MS metabolomics approach to identify metabolites in plasma samples collected from a total of 197 participants with or without cancer. We chose the untargeted metabolomic profiling approach as it is unbiased and allows for the discovery of novel targets associated with cancer‐related fatigue. We found that metabolites overrepresented in pathways, including sphingolipid metabolism, histidine metabolism, and cysteine and methionine metabolism, sufficiently distinguished patients with cancer‐related fatigue from healthy controls. Interestingly, even though fatigue levels were similar between non‐fatigued cancer patients and healthy controls, the two groups were metabolically distinct. By examining significant metabolites that are (1) unique to fatigued cancer patients, (2) associated with, but not unique to, fatigued cancer individuals (i.e., overlap pathways), and (3) associated with cancer but not fatigue, we provided a broad view of the metabolic phenotype of cancer‐related fatigue.

Sphingolipid metabolism, histidine metabolism, as well as cysteine and methionine metabolism were found to be specifically associated with fatigued, but not non‐fatigued, in cancer patients compared to healthy controls. Similar to recent findings in other diseases with fatigue symptoms such as myalgic encephalomyelitis/chronic fatigue syndrome (ME/CFS) and Gulf War Illness,^24^, ^25^ sphingolipid metabolism was the most significantly overrepresented metabolic pathway associated with fatigue in cancer patients (Figure 5B). Interestingly, we found elevated levels of the sphingomyelinase product, ceramide, in addition to the ceramide metabolite, sphingosine‐1‐phosphate in cancer patients who reported fatigue (Table S2). Excessive production of ceramide may lead to increased membrane permeability, altered mitochondrial calcium homeostasis, reactive oxygen species (ROS) generation, apoptosis, and enhanced inflammation.^27^, ^28^ It is possible that sphingolipid metabolism acts as an upstream regulator of prolonged inflammatory cytokine production that we previously found to be associated with cancer‐related fatigue.^7^, ^8^, ^9^ In addition to sphingolipid metabolism, albeit to a lesser degree, histidine metabolism, and cysteine and methionine metabolism were associated with fatigue in cancer patients. Interestingly, altered plasma levels of amino acids including histidine, cysteine, and methionine were observed in patients with chronic fatigue syndrome, task‐induced mental fatigue in healthy volunteers, as well as fatigue induced by forced swimming in rats.^29^, ^30^, ^31^ The change in plasma levels of histidine, cysteine, and methionine metabolites may be related to processes such as consumption of precursor amino acids and their conversion to antioxidants or neurotransmitters.^31^ Future studies will utilize radiolabeled amino acids and PET imaging to further investigate the role of amino acid metabolism and cancer‐related fatigue.

Pathways associated with, but not specific to, cancer‐related fatigue (overlap region of the Venn diagram in Figure 5A) included caffeine metabolism, tyrosine metabolism, steroid hormone biosynthesis, sulfur metabolism, and phenylalanine metabolism. Although caffeine is commonly known as the most popular central nervous system stimulant used to promote wakefulness,^32^ previous epidemiologic studies demonstrated an inverse association of coffee consumption with the progression of several types of cancer as well as all‐cause and cause‐specific mortality.^33^, ^34^ It is conceivable to postulate that caffeine metabolism is involved in both cancer‐related fatigue and cancer pathophysiology in general. However, it is worth noting that the involvement of caffeine metabolism may be difficult to interpret as data on caffeine intake by the study participants were not available. Future studies will investigate whether levels of caffeine metabolites adjusted for coffee intake are associated with fatigue in cancer patients. The other pathways associated with, but not unique to, cancer‐related fatigue (tyrosine metabolism, steroid hormone biosynthesis, sulfur metabolism, and phenylalanine metabolism) appear to be altered in several types of cancers and are associated with tumorigenesis.^35^, ^36^, ^37^, ^38^, ^39^ It is not clear what roles these metabolic pathways play in the pathogenic process of cancer‐related fatigue, though alterations in tyrosine metabolism, steroid hormone biosynthesis, and phenylalanine metabolism have been observed in chronic fatigue syndrome.^30^, ^40^ It is possible that pathways associated with, but not unique to, cancer‐related fatigue are involved in both fatigue pathogenesis and tumorigenicity due to their involvement in multiple processes.

Pathways related to only cancer, but not cancer‐related fatigue, included inositol phosphate metabolism, primary bile acid synthesis, ascorbate and aldarate metabolism, starch and sucrose metabolism, and pentose and glucuronate interconversions. In particular, inositol phosphate metabolism has been shown to regulate cancer motility, invasiveness, metastasis, and cancer pathogenicity in a variety of human cancers.^41^, ^42^ For example, inositol polyphosphate phosphatase 1 was shown to be highly expressed in aggressive human cancer cells and primary high‐grade human tumors.^41^ Interestingly, both bile acid metabolism and ascorbate and aldarate metabolism are among the metabolic pathways that exhibited highest expression heterogeneity in human cancer cell lines.^43^ Pentose and glucuronate interconversion is a metabolic pathway altered across all tumor types.^44^ Starch and sucrose metabolism genes were shown to be highly upregulated in metastatic cancer cell lines.^45^ The presence of these metabolic signatures suggests that cancer patients can be metabolically distinguished from healthy controls in the absence of any difference in reported fatigue severity.

One potential caveat is that plasma samples used in this study were collected from a mixed cancer population as our goal was to explore the general metabolic profile associated with the fatigue symptom in cancer patients. Future studies will explore metabolomic profiles associated with cancer‐related fatigue in specific types of cancer. Furthermore, we only include male participants as gender has been previously shown to affect metabolites associated with fatigue.^26^ We are currently recruiting female participants in order to compare metabolomic profiles of cancer‐related fatigue between genders. Participants enrolled in the study as healthy volunteers were younger than the cancer population. However, age did not correlate with fatigue severity or BMI. Age was also not significantly different between fatigued and non‐fatigued cancer patients. Therefore, we did not exclude younger healthy controls as age likely did not contribute significantly to metabolomic profiles associated with fatigue. Future studies will further examine metabolic profiles stratified by age with a larger sample size. We chose not to stratify the healthy controls based on their fatigue status because only three participants in the control group were considered fatigued. Future studies with a larger sample size will explore the mechanism of fatigue in healthy individuals. Interestingly, BMI was the highest in the fatigued cancer group compared to non‐fatigued cancer and healthy controls with no significant difference in BMI between non‐fatigued cancer and control groups. It is possible that reduced physical activity as a result of cancer‐related fatigue may contribute to the higher BMI in the fatigued cancer group. We are currently collecting actigraphy data in order to test the contribution of physical activity in fatigue. Lastly, as a natural history study that recruits all participants who met the inclusion criteria, the control group was not matched to the cancer group and the sample sizes of the groups were unequal. A retrospective power analysis was performed on all metabolites comparing the cancer cohort means against the control group mean via a Satterthwaite‐adjusted Student's two‐sample *t*‐test to accommodate circumstances where the variability in the groups might warrant its application (i.e., heteroscedasticity). The median power for the 1120 metabolite comparisons was 75% when conducted at the 0.05 level of statistical significance (two‐sided test), and 84% at a significance threshold of 0.10 (see Lehmann 2005, for a commentary on the choice of statistical significance level based on context), which is acceptable as the nominal level of power used is 80% in many confirmatory studies.^46^

In conclusion, results from this global metabolomics study using plasma samples collected from cancer patients with or without fatigue compared to healthy controls revealed significant metabolomic profile differences associated with cancer‐related fatigue. The predictive models generated in the study predicted group classification at a high degree of accuracy. Potentially impactful metabolic pathways associated specifically with fatigued cancer patients include changes in metabolites associated with sphingolipid metabolism, histidine metabolism, and cysteine and methionine metabolism.

## CONFLICT OF INTEREST

The authors have no conflict of interest to declare.

## AUTHOR CONTRIBUTION

All authors meet the criteria for authorship as defined by the ICMJE definition of authorship.

## Supporting information

Table S1

Table S2

Table S3

## Data Availability

All metabolomics data for “Plasma metabolomic profile associated with fatigue in cancer patients” was deposited in the Open Science Framework database (https://osf.io/) under the DOI number https://doi.org/10.17605/OSF.IO/DTUKV.
