# Supplementary material for: Plasma metabolomic profile associated with fatigue in cancer patients
Source: Cancer Med. 2021 Feb 3;10(5):1623–33. doi: 10.1002/cam4.3749 (PMC7940245; doi:10.1002/cam4.3749)
Supplement: Supplementary file 1 — Table S1 [file CAM4-10-1623-s003.docx]

**Supplemental Table 1**. Demographics and clinical characteristics of sample population.

|  | **Cancer**  **Fatigued**  **(n = 49)** | **Cancer**  **Non-fatigued**  **(n = 122)** | **Healthy**  **Controls**  **(n = 26)** |  |
| --- | --- | --- | --- | --- |
| **Age (years)** | 65.35 ± 8.58 | 65.25 ± 8.55 | 35.92 ± 15.03 |  |
| **BMI (kg/m^2^)** | 30.37 ± 5.34 | 27.85 ± 3.93 | 26.81 ± 3.65 |  |
| **Race/Ethnicity**  *Asian*  *African American*  *Hispanic*  *Caucasian*  *Other* | 4.08%  16.3%  2.04%  77.6%  0.00% | 5.74%  22.1%  2.46%  69.7%  0.00% | 3.85%  19.2%  7.69%  61.5%  7.69% |  |
| **Cancer Type**  *Prostate Adenocarcinoma, non-metastatic*  *Prostate Adenocarcinoma, metastatic*  *Prostatic Adenocarcinoma (ductal), non-metastatic*  *Choroidal Tumor, non-metastatic*  *Epithelioid Uveal Melanoma Liver, metastatic*  *Lung Adenocarcinoma, non-metastatic*  *Colon Adenocarcinoma, metastatic*  *Pancreatic Adenocarcinoma, metastatic*  *Ocular Melanoma, non-metastatic*  *Urothelial Carcinoma, non-metastatic* | 85.7%  10.2%  0.0%  0.0%  0.0%  2.0%  2.0%  0.0%  0.0%  2.0% | 95.9%  1.6%  0.8%  0.8%  0.8%  0.0%  0.0%  0.8%  0.8%  0.0% | N/A |  |
| **T-stage**  *T0*  *T1c*  *T2a-c*  *T3a-c*  *T4*  *Tx* | 6.12%  28.6%  28.6%  22.5%  8.16%  6.12% | 4.10%  36.9%  34.4%  22.1%  0.82%  1.64% | N/A | |

Abbreviations: BMI, body mass index; CBC, Complete Blood Count; g/dL, grams per deciliter; Hb, hemoglobin; kg/m^2^, kilogram per meter squared; K/uL, thousand per cubic milliliter; M/uL, million per microliter; NA, Not Applicable; ng, nanogram; RBC, red blood cells; WBC, white blood cells.
